# Identification of Maize Kernel Varieties Using LF-NMR Combined with Image Data: An Explainable Approach Based on Machine Learning

**DOI:** 10.3390/plants14010037

**Published:** 2024-12-26

**Authors:** Chunguang Bi, Xinhua Bi, Jinjing Liu, He Chen, Mohan Wang, Helong Yu, Shaozhong Song

**Affiliations:** 1 Institute for the Smart Agriculture, Jilin Agricultural University, Changchun 130118, China; chunguangb@jlau.edu.cn; 2 College of Information Technology, Jilin Agricultural University, Changchun 130118, China; 20231269@mails.jlau.edu.cn (X.B.); 20231616@mails.jlau.edu.cn (J.L.); 20221125@mails.jlau.edu.cn (H.C.); 3 Zhongnong Sunshine School-Enterprise R&D Centre, Jilin Agricultural University, Changchun 130118, China; wangmohan@znygsj.com; 4 School of Data Science and Artificial Intelligence, Jilin Engineering Normal University, Changchun 130052, China

**Keywords:** maize kernels, LF-NMR, multi-source data, SVM, Shapley value, germplasm resource

## Abstract

The precise identification of maize kernel varieties is essential for germplasm resource management, genetic diversity conservation, and the optimization of agricultural production. To address the need for rapid and non-destructive variety identification, this study developed a novel interpretable machine learning approach that integrates low-field nuclear magnetic resonance (LF-NMR) with morphological image features through an optimized support vector machine (SVM) framework. First, LF-NMR signals were obtained from eleven maize kernel varieties, and ten key features were extracted from the transverse relaxation decay curves. Meanwhile, five image morphological features were selected using the recursive feature elimination (RFE) algorithm. Before modeling, principal component analysis (PCA) was used to determine the distribution features of the internal components for each maize variety. Subsequently, LF-NMR features and image morphological data were integrated to construct a classification model and the SVM hyperparameters were optimized using an improved differential evolution algorithm, achieving a final classification accuracy of 96.36%, which demonstrated strong robustness and precision. The model’s interpretability was further enhanced using Shapley values, which revealed the contributions of key features such as Max Signal and Signal at Max Curvature to classification decisions. This study provides an innovative technical solution for the efficient identification of maize varieties, supports the refined management of germplasm resources, and lays a foundation for genetic improvement and agricultural applications.

## 1. Introduction

Global food security is becoming increasingly challenging, and maize, as one of the world’s major food crops [1], shoulders an important mission of ensuring the world’s food supply. With the crossbreeding and development of different maize varieties, the decline in seed purity has not only affected crop yields but also restricted the improvement of agricultural production efficiency [2,3,4]. Traditional methods for identifying maize varieties rely mainly on manual experience and simple physical feature analysis, such as morphological methods, protein electrophoresis, and DNA molecular marker technology [5,6,7]. These methods are inefficient when dealing with large-scale or complex data, have limited accuracy, and are susceptible to subjective factors. Therefore, accurate and rapid identification of corn varieties not only helps to improve the efficiency of agricultural production, but also ensures that high-quality seeds enter the market, reduces waste of resources, and improves the sustainability of agricultural production [8]. Most existing machine learning models for variety identification lack interpretability, making it difficult to understand the biological basis of their decisions. The optimization of model parameters often relies on simple algorithms that may not fully capture the complex relationships in multi-source data.

Machine vision technology has been widely used in corn seed classification. It can effectively identify different varieties of corn seeds by analyzing their external morphological characteristics, such as color and texture [9,10,11]. However, machine vision has limited ability to identify internal chemical composition. Studies have used hyperspectral imaging to obtain rich spectral information from seeds for analysis of their chemical composition, providing more detailed results than traditional methods [12,13,14]. Nevertheless, hyperspectral imaging relies on spectral reflectance to infer internal composition, which limits its ability to analyze the internal structure and complex composition of seeds. To overcome this challenge, LF-NMR provides another viable non-destructive testing method. By measuring the relaxation times (T_1_ and T_2_) of hydrogen protons, LF-NMR can provide detailed information about the internal structure of the sample, and T_2_ is gradually becoming more popular among researchers due to its higher sensitivity and shorter acquisition time [15]. In recent years, LF-NMR technology has been widely used in fields such as biomedicine [16,17], petroleum and energy [18,19], and materials science [20,21]; there are other fields that it was been widely used in, especially in field of food and agriculture [22], and it has been used for edible oil adulteration detection [23,24], fruit juice classification [25], and corn hardness prediction [26], demonstrating significant research value and application potential. Recent advances in non-destructive testing have demonstrated the potential of combining low-field nuclear magnetic resonance (LF-NMR) with machine learning techniques for improved detection and classification. For instance, Fu et al. [27] combined LF-NMR with deep learning neural networks (DLNN) to successfully detect defects in dried longan, achieving an accuracy of 89%. Zhao et al. [28] employed the SE_AlexNet_MiniConv model for defect detection in ginkgo seeds, attaining an impressive accuracy of 96.92%. Song et al. [29] integrated LF-NMR with multispectral imaging (MSI) technology, significantly enhancing the classification accuracy of wheat seedlings under salt stress, with a Gaussian Naive Bayes model yielding an accuracy of 88.90%. Additionally, Ribeiro et al. [30] demonstrated the effectiveness of low-frequency 1H NMR technology in distinguishing between different plant sources for honey classification, showing significant correlations with T_2_ parameters. However, most existing machine learning models for variety identification lack interpretability, making it difficult to understand the biological basis of their decisions. Additionally, the optimization of model parameters often relies on simple algorithms that may not fully capture the complex relationships in multi-source data.

Table 1 shows the results of different group intelligence algorithms optimizing machine learning hyperparameters. Compared with traditional optimization algorithms, group intelligence optimization algorithms have demonstrated superior performance in determining the best hyperparameters [31]. In addition, explainable artificial intelligence and machine learning have been widely used in many fields [32], providing more transparent and efficient solutions. In the field of agriculture, explainable models play an important role. One study developed an explainable ensemble model for wheat grain classification, combining SHAP to improve the interpretability of the model, and ultimately achieving a classification accuracy of 94% [33]. In corn yield prediction, researchers used a multimodal deep learning model combining RGB images, phenotypes, and meteorological data, achieving a prediction accuracy of 89%. SHAP analysis was used to identify the key factors affecting yield, providing an important reference for agricultural management [34]. Raman spectroscopy combined with an XGBoost model was used for cottonseed variety identification, achieving a classification accuracy of 0.88 to 0.94. The study also found that lignin is an important feature for classification through SHAP analysis [35]. These studies have provided critical support for the development of agricultural production efficiency and crop variety identification technology, laying a theoretical and practical foundation for the subsequent in-depth application of maize variety identification technology.

This study aims to provide technical support for the optimization and conservation of maize germplasm resources by proposing a novel interpretable machine learning method for maize kernel varieties identification that integrates LF-NMR and image morphological data. The specific objectives of the study are (1) effectively integrating average low-field NMR features with image morphological characteristics; (2) improving the accuracy of maize kernel variety identification through the application of an enhanced differential evolution algorithm; (3) incorporating the SHapley Additive exPlanations interpretability framework, which enhances model transparency and ensures that the identification results are both accurate and interpretable.

## 2. Materials and Methods

### 2.1. Material Preparation

**Figure 1:**
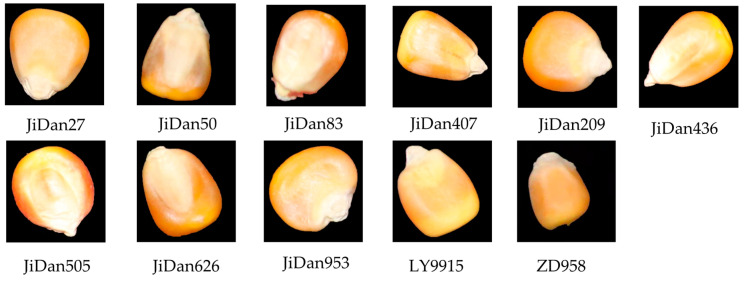
Maize kernel samples.

The maize seed samples used in this study were provided by the Institute of Smart Agriculture at Jilin Agricultural University and included a total of 11 varieties: JiDan27, JiDan50, JiDan83, JiDan209, JiDan407, JiDan436, Ji Dan505, JiDan626, JiDan953, LY9915, and ZhengDan958 (Figure 1). The internal chemical composition of each variety is detailed in Table 2. All selected seeds are yellow, with a few varieties having a slightly reddish surface. In order to ensure the purity and integrity of the samples, this study preferred yellow-colored seeds with some reddish hues, and damaged, worm-eaten, and impure seeds were removed by hand. The final sample of full and intact seeds was 1000 grains of each variety.

### 2.2. Data Acquisition

#### 2.2.1. Image Data Acquisition

**Figure 2:**
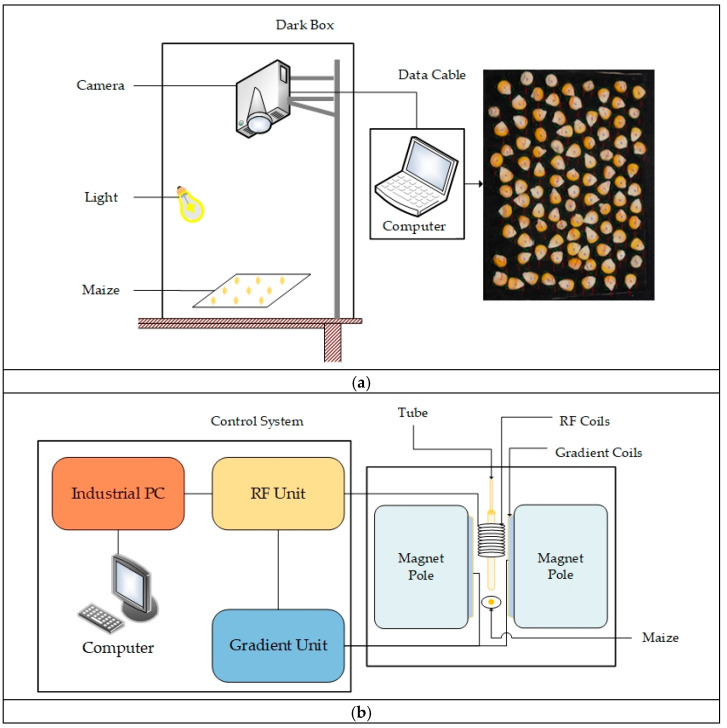
Schematic diagram of data acquisition: (**a**) image data; (**b**) LF-NMR data.

Maize kernels images were taken with a Canon EOS 1500D camera produced by Canon, located in Tokyo, Japan. To ensure stability and consistency in the data acquisition environment, all images were captured under controlled conditions to avoid interference from external light sources and minimize the impact of external factors on image quality. Seeds were placed on a black background plate, with the camera mounted vertically above it, and two stabilized LED light sources were used to provide consistent illumination. Figure 2a shows the arrangement of the collection equipment. The seeds of each variety were arranged in groups of 100, and a total of 10 sets of images were taken, each with a resolution of 6000 × 4000 pixels.

#### 2.2.2. LF-NMR Data Acquisition

In this study, a low-field nuclear magnetic resonance analyzer (Model: MesoMR23-060H-I) from Newmax Electronic Technology Co., Ltd., Shanghai, China was used, equipped with a NIMI20-015V-I magnet probe, which has a magnetic field strength of 0.5 T and a coil diameter of 15 mm. A schematic diagram of the acquisition setup is presented in Figure 2b. Thirty seeds from each maize variety were randomly selected for measurement. The instrument was recalibrated before each measurement to ensure data accuracy and consistency. To minimize the influence of ambient light on the results, all experiments were conducted in a controlled laboratory environment.

### 2.3. Experimental Procedure

The computer environment used in this study was as follows: CPU—Intel(R) Xeon(R) Gold 6246R CPU @ 3.40 GHz; RAM—128 GB; GPU—NVIDIA Quadro RTX 8000; 64-bit Windows 10 operating system; Python version 3.8.

**Figure 3:**
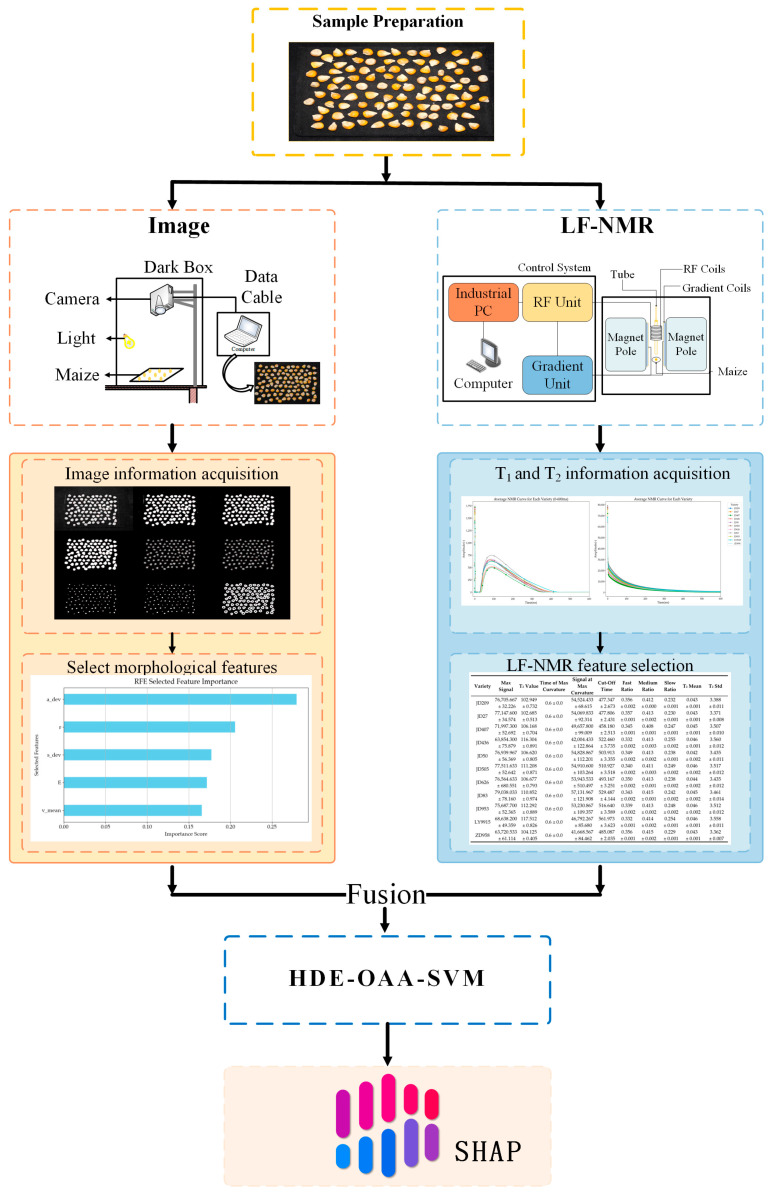
Experimental flow chart.

The experimental method used in this study is shown in Figure 3. First, the morphological feature data were extracted from the RGB image using a ‘machine vision-based reference-free maize kernel phenotype measurement system’ and combined with the LF-NMR feature data. Then, PCA was performed on the decay curve features, and the classification order was adjusted to optimize the classification effect by analyzing the distribution of maize seeds in the principal component graph. Subsequently, an improved differential evolution algorithm was used to optimize the model parameters. Finally, an interpretable method was used to analyze the decision-making process of the model.

### 2.4. Feature Selection

Image morphological feature extraction aims to extract geometric, texture, and color information related to its physical form from maize kernel images. Firstly, the image is analyzed after segmentation using the watershed algorithm, the contour of each kernel is obtained by a boundary tracking algorithm, and geometric features, including area, perimeter, and aspect ratio, are calculated. In addition, texture features such as grey level covariance matrix (GLCM) features are extracted for describing the contrast, similarity, and uniformity of the image. Color features are obtained by analyzing the RGB color space and the histogram of color distribution. Each feature provides unique information that is used to accurately distinguish between different maize varieties. In this study, the recursive feature elimination (RFE), mutual information (MI), and ReliefF algorithms are used for image feature selection.

The LF-NMR feature extraction aims to obtain important information from the acquired NMR data that can help in variety identification. Transverse relaxation decay curve data were acquired during the measurements to record the signal amplitude of hydrogen protons at various time points when excited by a 180° phase inversion pulse of the CPMG sequence. The data were processed using Savitzky–Golay (SG) smoothing, which reduces noise while retaining key features of the signal, providing a truer representation of the chemical and physical properties of the sample. The data include T_2_ relaxation times and corresponding inversion curves, which provide the basis for subsequent data analysis and feature extraction. In order to visualize the different signal amplitudes of different maize varieties at different times, Pycharm2021.3.3 software was used for graphical display. Specifically, Maximum Signal (a.u.), T_2_ Value (ms), Time Point of Maximum Curvature (ms), Signal Corresponding to Maximum Point of Curvature (a.u.), Signal Cut-off Time (ms), fast_ratio, medium_ratio, slow_ratio, T_2__mean, and T_2__std were used.

### 2.5. Data Analysis and Modeling Methods

#### 2.5.1. Principal Component Analysis (PCA)

PCA is widely used as a convenient data extraction and dimensionality reduction tool in the analysis of high-dimensional covariate data samples [41]. Principal component analysis was performed on a total of 330 samples to visually obtain the relationship between different corn varieties and provide a basis for classification by the SVM classification model. First, a matrix was created with the varieties of maize seeds as rows and the five characteristics of maize as columns to represent the sample information for each variety, and this matrix was used as the input variable for the preprocessing program. The results included a covariance matrix, eigenvalues, and a corresponding eigenvector matrix (principal component scores). Based on the eigenvalues, the contribution rate of each component and its cumulative value can be determined. By analyzing the score values of each component, the distance and relative position between different varieties can be clearly displayed.

#### 2.5.2. Improved Differential Evolution Algorithm

For multiclassification tasks, it is common to construct multiclass classifiers by combining multiple binary classifiers. The classification method used in this paper is based on the One-against-all support vector machine algorithm and uses five-fold cross-validation to improve the stability of the algorithm [42]. To improve the performance of support vector machine models, a differential evolution algorithm is used to optimize hyperparameters [43]. However, traditional differential evolution algorithms have some limitations in practical applications, which limits their optimization performance and convergence speed. This paper proposes the following two main improvements to improve the performance of the differential evolution algorithm in optimizing support vector machine models:

First, an adaptive control mechanism that dynamically adjusts the mutation factor and recombination rate; the mutation factor (F) and recombination rate (CR) are key parameters in differential evolutionary algorithms that directly affect the exploration and exploitation capabilities of the algorithms. Fixed variance factor and recombination rate may perform poorly in different optimization stages. In this paper, a dynamic adjustment mechanism is proposed so that these two parameters can be adjusted according to the convergence in the optimization process to improve the adaptability and performance of the algorithm. Let the current iteration number be t and the convergence situation be $c_{t}$; then, the dynamic adjustment formula is as follows:

(1) $$F_{t+1}=\left\{\begin{array}{c} 0.2+0.5\cdot r,\;c_{t}\leq 0.05\\ 0.3+0.5\cdot r,\;0.05\;{<c}_{t}\leq 0.1\\ 0.5+0.5\cdot r,\;c_{t}>\;0.1 \end{array}\right.$$

(2) $$CR_{t+1}=\left\{\begin{array}{c} 0.95,\;c_{t}\leq 0.05\\ 0.9,\;0.05\;{<c}_{t}\leq 0.1\\ 0.7,\;c_{t}>\;0.1 \end{array}\right.$$

where r is a random number in the range [0, 1] used to introduce randomness to avoid premature convergence. With this dynamic adjustment mechanism, the algorithm has strong global search capabilities in the early stages, and in the later stages, it focuses more on enhancing local search capabilities, thereby improving the overall optimization effect. Meanwhile, the adaptive control mechanism dynamically adjusts the parameters by monitoring the diversity and convergence of the population. Let the diversity of the current population be $D_{Current}$, and the maximum diversity be $D_{max}$; then, the adjustment formula for the variation factor and recombination rate is

(3) $$F_{t+1}=F_{base}+∆F\cdot \left(1-\frac{D_{Current}}{D_{max}}\right)$$

(4) $$CR_{t+1}=CR_{base}+∆CR\cdot \left(1-\frac{D_{Current}}{D_{max}}\right)$$

where $F_{base}$ and ${CR}_{base}$ are the baseline variance factors and recombination rates, and ∆F and ∆CR are the adjustment margins. Through this adaptive control mechanism, the algorithm can automatically adjust the parameters at different optimization stages to improve the overall performance and convergence speed.

Second, the combination of multiple mutation strategies: the mutation strategies used in differential evolution algorithms directly affect the diversity of the population and the algorithm’s global search ability. Traditional differential evolution algorithms usually use a single mutation strategy, such as DE/rand/1/bin or DE/best/1/bin. Such a single strategy may not be sufficient to provide enough diversity in some cases, which results in the algorithm easily falling into local optimal solutions. Therefore, this paper proposes to combine multiple variation strategies to enhance the algorithm’s global search and local exploitation capabilities including rand1, best1, current-to-best1, and best2. The variation strategy formulas are as follows:

DE/rand/1/bin strategy:

(5) $$v_{i}^{\left(t+1\right)}=x_{r1}^{\left(t\right)}+F\cdot \left(x_{r2}^{\left(t\right)}-x_{r3}^{\left(t\right)}\right)$$

where $x_{r1}^{\left(t\right)}$, $x_{r2}^{\left(t\right)}$, and $x_{r3}^{\left(t\right)}$ are three different individuals chosen at random.

DE/best/1/bin strategy:

(6) $$v_{i}^{\left(t+1\right)}=x_{best}^{\left(t\right)}+F\cdot \left(x_{r1}^{\left(t\right)}-x_{r2}^{\left(t\right)}\right)$$

where $x_{best}^{\left(t\right)}$ is the current optimal individual; $x_{r1}^{\left(t\right)},\;x_{r2}^{\left(t\right)}$ are two different individuals chosen at random.

DE/current-to-best/1 strategy:

(7) $$v_{i}^{\left(t+1\right)}=x_{i}^{\left(t\right)}+F\cdot \left(x_{best}^{\left(t\right)}-x_{i}^{\left(t\right)}\right)+F\cdot \left(x_{r1}^{\left(t\right)}-x_{r2}^{\left(t\right)}\right)$$

where $x_{i}^{\left(t\right)}$ is the current individual; $x_{best}^{\left(t\right)}$ is the current optimal individual; $x_{r1}^{\left(t\right)}$, $x_{r2}^{\left(t\right)}$ is two different individuals chosen at random.

DE/best/2/bin strategy:

(8) $$v_{i}^{\left(t+1\right)}=x_{best}^{\left(t\right)}+F\cdot \left(x_{r1}^{\left(t\right)}-x_{r2}^{\left(t\right)}\right)+F\cdot \left(x_{r3}^{\left(t\right)}-x_{r4}^{\left(t\right)}\right)$$

where $x_{best}^{\left(t\right)}$ is the current optimal individual, and $x_{r1}^{\left(t\right)}$, $x_{r2}^{\left(t\right)}$, $x_{r3}^{\left(t\right)}$, and $x_{r4}^{\left(t\right)}$ are four different individuals chosen at random.

The above variant strategies have different advantages at different stages of optimization. The main goal of the initial phase is to explore the search space extensively to find possible high-quality solutions, so strategies such as DE/rand/1/bin are suitable to increase the population diversity. The intermediate stage requires finding a balance between exploring new solutions and exploiting existing ones, so strategies such as DE/current-to-best/1 can be used, while in the later stages, the main goal is to fine-tune the exploitation of the better solutions, so strategies such as DE/best/1/bin and DE/best/2/bin are more suitable. By combining multiple variant strategies, the global search and local development ability of the algorithm can be enhanced while maintaining the diversity of the population.

The pseudo-code implementation of the improved differential evolution (HDE) is shown in Table 3.

## 3. Results

### 3.1. Feature Analysis

**Figure 4:**
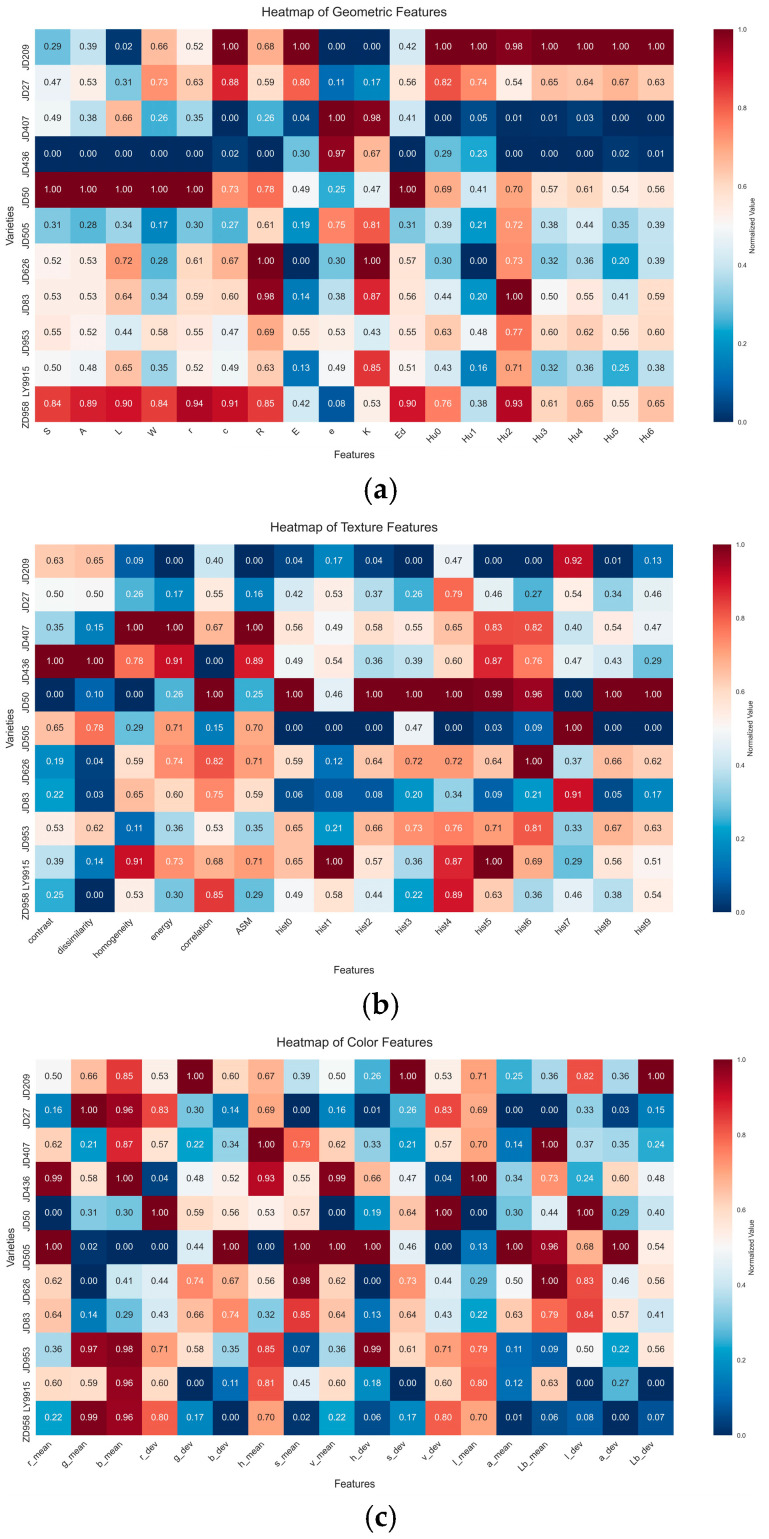
Heatmap of the morphological features of different types of kernels: (**a**) geometric features, (**b**) textural features, (**c**) color features.

**Figure 5:**
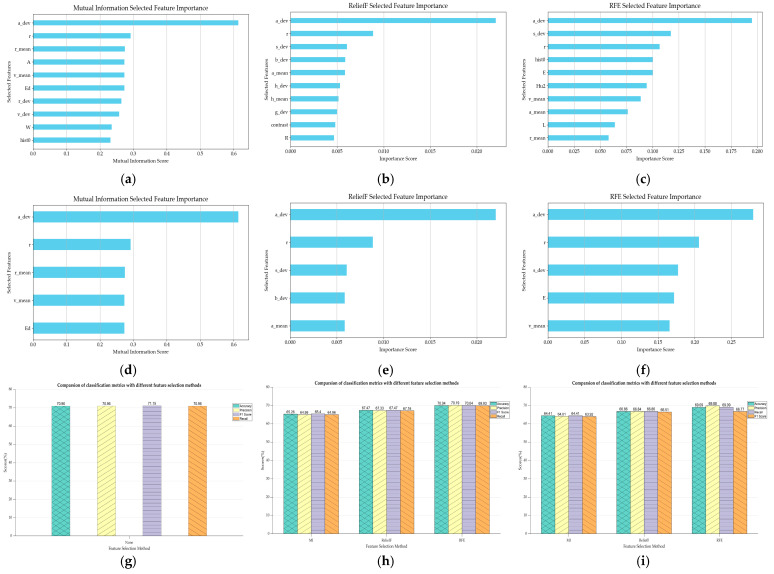
Results using feature selection: (**a**–**c**) MI, ReliefF, RFE to select 10 features; (**d**–**f**) MI, ReliefF, RFE to select 5 features; (**g**–**i**) comparison of identification results.

This paper extracted 52 morphological features from corn seeds, and the heatmap of normalized average values of different seed categories are shown in Figure 4. The analysis of shape feature distributions in Figure 4a reveals significant differences between categories. Among the features, ‘E’ and ‘r’ exhibit greater dispersion, indicating that these features have high variability across different corn kernels and possess strong discriminative power. Figure 4b displays the mean value distribution of the texture features, where it is observed that JD436 differs significantly from the other categories in the contrast feature, while JD50 shows notable distinction in the hist0 feature. Figure 4c shows the mean distribution of color features. Different varieties showed significant differences in color features, especially in ‘g_mean’ and ‘b_mean’, and several varieties showed obvious differentiation. In terms of color deviation characteristics, JD209 has obvious characteristics in the ‘g_dev’ characteristic, while JD505 shows high values in the ‘a_dev’ characteristic. A number of varieties also showed large differences in ‘s_dev’ and ‘v_dev’, which are important for variety identification. Three feature selection algorithms were applied to select ten and five features from fifty-two, and their corresponding accuracy rates were compared, as shown in Figure 5. The accuracy rates for the ten and five features selected using RFE were 70.04% and 69.09%, respectively, both of which outperform the ReliefF and MI algorithms. Furthermore, the accuracy of the five features selected by RFE is only 1.87% lower than that of the model without feature selection, while the number of features is reduced by a factor of 9.4. Based on these results, the RFE algorithm was selected for the subsequent experiments in this study.

Among the selected morphological features, ‘v_mean’ and ‘s_dev’ represent the mean value and standard deviation of the saturation of the seed in the HSV color space, respectively, while ‘a_dev’ is the standard deviation of the red channel in the RGB color space. In addition, ‘r’ measures the compactness of the seed shape, indicating how flat the seed shape. ‘E’ is represented by the ratio of the length of the short axis to the length of the long axis of the seed. The above features not only comprehensively reflect the morphological characteristics of maize seeds from multiple dimensions such as shape, texture, and color, but also provide a scientific basis for further classification and quality analysis.

**Figure 6:**
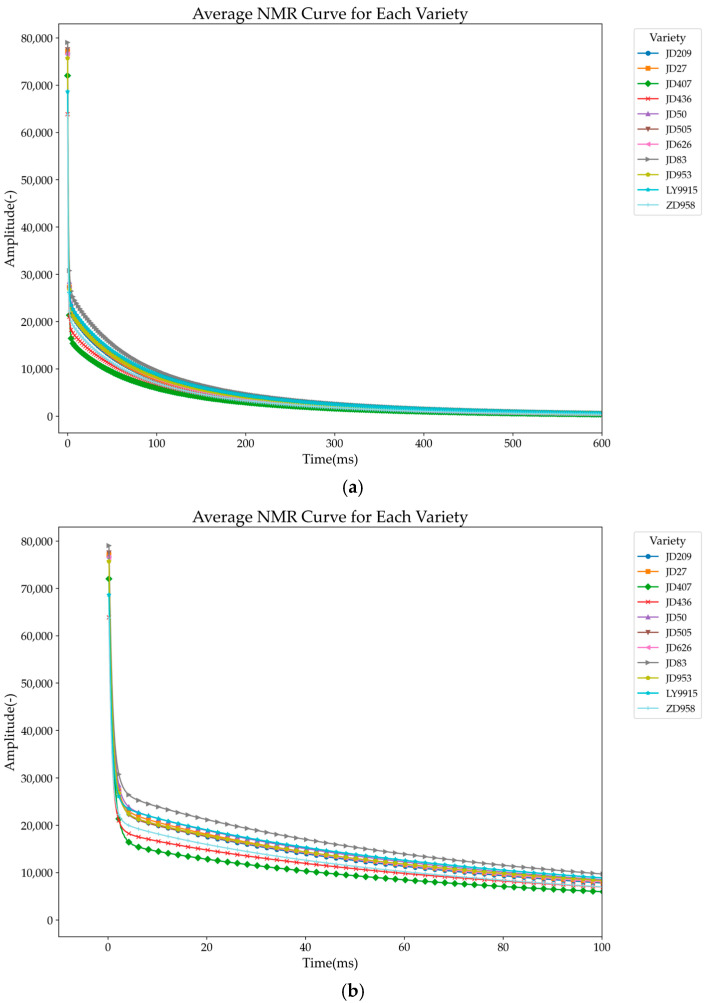
Average T_2_ relaxation time curves for eleven maize varieties: (**a**) 0–600 ms; (**b**) 0–100 ms.

Figure 6 shows the T_2_ decay curves of 11 maize varieties. Although the overall decay trend of these varieties is similar, there are significant differences in the decay rate. For example, the grey right triangle curve represents JD83, which decays the slowest, indicating that JD83 has the longest echo decay time; in contrast, the green diamond curve represents JD407, which decays the fastest and is the first variety to reach the end of decay. The decay curves of the other varieties fall between JD83 and JD407.

In order to obtain clearer and more intuitive information about the T_2_ decay curve, the characteristics of the sample were extracted from the transverse relaxation decay curve. The average time was 15.92 s, which was more efficient than the traditional method. The results are summarized in Table 4, and the data are presented as the mean ± standard deviation. Table 3 shows that the extracted features of the 11 maize varieties are significantly different. The maximum signal value reflects the ability of the sample to respond to the magnetic pulse, which is related to the number and state of the protons. JD83 has the highest maximum signal (79,038.033 ± 78.160 a.u.), while ZD958 has the lowest (63,720.533 ± 61.114 a.u.). These differences reflect the different moisture binding states and proton concentration of the different varieties. The T_2_ value and signal truncation time indicate the time for hydrogen protons to decay to 37% and 0% of the maximum signal, respectively. These two parameters reflect the T_2_ decay rate of hydrogen protons in the sample: the smaller the T_2_ value and the shorter the signal truncation time, the faster the echo attenuation. In corn seeds, the T_2_ value is mainly affected by the crude protein, crude fat, and crude starch content. A higher crude fat content reduces the free mobility of water, which in turn prolongs the T_2_ value, while a higher crude protein content enhances the interaction between water molecules and proteins, shortening the T_2_ value. An increase in the crude starch content leads to a denser seed structure, which accelerates the attenuation of the echo signal. For example, the JD407 has the shortest T_2_ value (106.168 ± 0.704 ms) and signal end time (458.180 ± 2.513 ms), indicating the fastest relaxation rate, which is consistent with its highest crude starch content (76.60%). In contrast, JD83 had the largest T_2_ value (110.852 ± 0.974 ms) and signal end time (529.487 ± 4.144 ms), possibly because its high crude protein content (10.92%) restricted the free movement of water molecules. In addition, LY9915 had the highest signal end time of 561.973 ± 3.623 ms, which is consistent with its highest crude fat content (4.99%).

The other two features indicate the time and signal value corresponding to the point of maximum decay curvature, respectively. Most corn varieties reach the maximum curvature point at 0.6 ± 0.0 ms. JD505 and JD50 have higher signal values, 54,910.600 ± 103.264 a.u. and 54,828.867 ± 112.201 a.u., indicating a strong signal response, while JD407 had the lowest signal value, 49,657.800 ± 99.009 a.u., which is consistent with its low crude fat content and high crude starch content. In addition, ZD958 and JD436 showed slow decay and relatively low signal values at the maximum curvature point, 41,668.567 ± 84.462 a.u. and 42,004.433 ± 122.864 a.u., respectively, indicating unique relaxation characteristics. The Fast Ratio, Medium Ratio and Slow Ratio further feature the different water binding states. The Fast Ratio, Medium Ratio, and Slow Ratio further feature the different water binding states. Varieties with a high Fast Ratio (e.g., JD27 and JD209) contain more free water; varieties with a high Medium Ratio (e.g., LY9915 and JD83) show stronger medium-binding water action; and varieties with a high Slow Ratio (e.g., JD436 and LY9915) reflect stronger water binding. Finally, the T_2_ Mean and T_2_ Std provide overall information on the relaxation distribution. JD83 has a higher T_2_ Mean (0.045 ± 0.002), indicating slower relaxation, while JD50 has a lower T_2_ Mean (0.042 ± 0.002), indicating a faster relaxation process. Overall, these features reveal the quantity and state of hydrogen protons inside the corn seed from different perspectives, reflecting significant differences between varieties. Further chemometric analysis based on these features can provide more scientific evidence about seed composition and its interactions, thus providing data support for the study of the physiological characteristics of corn varieties.

In order to achieve an optimal balance between model performance and computational efficiency, this study conducted a comparative analysis of classification performance using the complete set of 3000 features versus a reduced set of 10 key features from LF-NMR data. The experimental results demonstrate that while the full-feature model achieved superior performance, it incurred substantial computational overhead due to its high dimensionality. In contrast, although the dimensionality-reduced model showed slightly decreased performance metrics, it maintained satisfactory classification effectiveness and stability, indicating the representative nature of the selected key features, as shown in Table 5. Considering the practical constraints of computational efficiency and model complexity in real-world applications, this study adopted the reduced 10-feature set for subsequent experiments, thereby significantly reducing computational complexity while maintaining model practicality.

**Figure 7:**
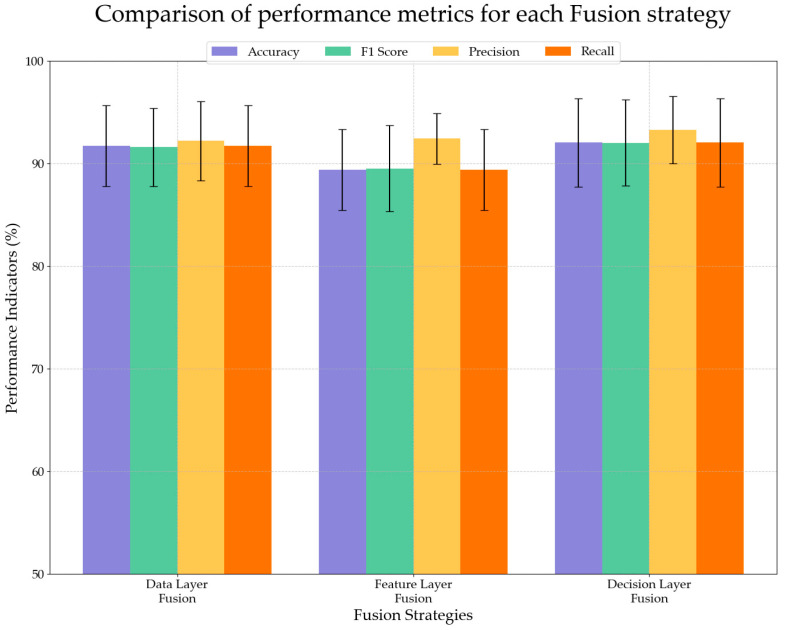
Comparison of performance metrics for each fusion strategy.

At present, multi-source data fusion methods are mainly divided into three categories: data layer fusion, feature layer fusion, and decision layer fusion [44]. As shown in Figure 7, feature layer fusion performs well in terms of accuracy (92.43% ± 2.47%) and has the smallest standard deviation, showing good stability. Although other metrics are slightly lower than data layer fusion (91.72% accuracy) and decision layer fusion (92.04% accuracy), feature layer fusion only needs to process 15 input features, which has a significant computational efficiency advantage over the 3053 samples processed by the other two methods. Considering the practical needs of model stability and computational efficiency, this study adopts the feature-level fusion method to integrate the LF-NMR data and image data of maize kernels to improve the prediction performance of the model. By combining the complementary characteristics of these two types of data to form a comprehensive feature space, the model can take advantage of both low-field nuclear magnetic resonance and image data, thereby improving the accuracy and robustness of maize kernel identification. This fusion method makes full use of the complementary nature of nuclear magnetic resonance data and image data in characterizing maize kernel quality and provides a practical reference for the application of multi-source data fusion.

### 3.2. Results of PCA

**Figure 8:**
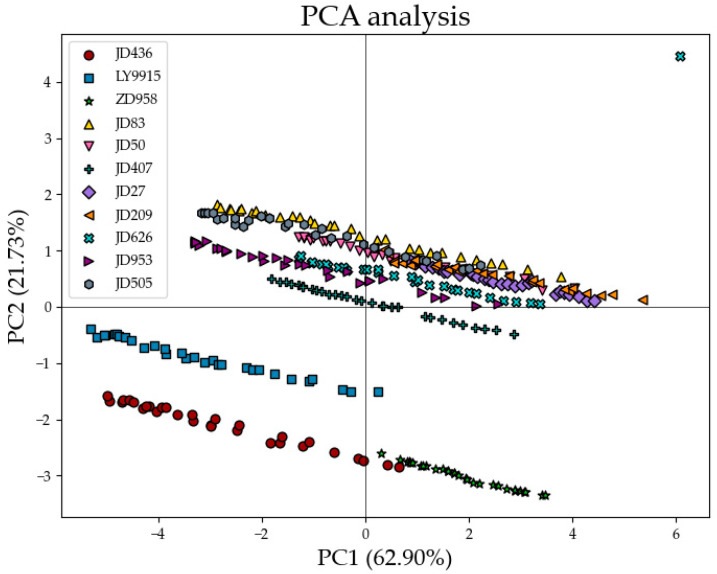
Scores of 11 types of maize kernels for the first two principal components.

Figure 8 shows the results of projecting all 11 maize varieties onto the first two principal components, which contribute 62.90% and 21.73%, respectively. The figure shows a clear tendency for the different maize varieties to cluster together. This result is highly consistent with the results of the features extracted from the T_2_ relaxation curve. The distribution in the figure is determined by the main component differences in the maize kernels. PC1 mainly reflects the differences in crude protein and crude starch content. For example, JD436 (crude protein 10.65%, crude starch 76.39%) is distributed in the negative direction of PC1, while ZD958 (crude protein 8.47%, crude starch 73.42%) and JD27 (crude protein 8.46%, crude starch 75.23%) are located in the positive direction of PC1, indicating that high crude protein and high starch varieties tend to cluster in the negative direction of PC1, while low crude protein and low starch varieties predominate in the positive direction. LY9915 (crude protein 10.58%, crude starch 73.3%) still clusters significantly in the negative direction of PC1 due to its high crude protein content, despite its moderate crude starch content. PC2 reflects differences in crude fat and lysine content. For example, varieties with high crude fat and lysine content (e.g., ZD958, with 3.92% crude fat and 0.37% lysine, and JD50, with 4.31% crude fat and 0.32% lysine) are clearly characterized in the positive direction of PC2, while the low-lysine variety (e.g., JD505, with 0% lysine) is located in the negative direction of PC2. In addition, the clustering distribution of different varieties further illustrates the influence of compositional similarity. For example, JD436 and JD407 form an aggregate in the negative direction of PC1, while ZD958 and JD27 show independence in the positive directions of PC1 and PC2. These distribution characteristics provide a clear statistical and biological explanation for the compositional differences between maize varieties, laying an important foundation for subsequent research on their quality and application potential. Subsequently, SVM was used to classify maize varieties into different categories. The classification results showed significant differences between categories, providing an important basis for further optimizing the selection and breeding strategies of maize varieties.

### 3.3. Model Performance Evaluation

This study first compares the performance of a variety of classification models, including OAA-SVM, logistic regression, random forest, K-nearest neighbors, MLP classifiers, and XGBoost, and comprehensively analyzes their classification accuracy, F1 score, precision, and recall rate. The results show that the OAA-SVM model performs well in all indicators, with an average classification accuracy of 89.39%, which is higher than that of other models, as shown in Table 6.

On this basis, the influence of LF-NMR data and image data fusion on classification performance was further explored. The experimental results show that when image data are used alone, the OAA-SVM model has a classification accuracy of 69.09% on the validation set, and when only low-field nuclear magnetic resonance data are used, the OAA-SVM model has a classification accuracy of 83.03% on the validation set. When image data are fused with nuclear magnetic resonance data, the classification accuracy increases to 89.39%, an increase of 6.36% compared to only LF-NMR. Furthermore, the DE-OAA-SVM model (Differential Evolution optimized One-vs-All Support Vector Machine) obtained by parameter optimization of OAA-SVM using the differential evolution algorithm improved the accuracy to 93.94%, an increase of 4.55% over OAA-SVM. Finally, using the improved differential evolution algorithm to further optimize HDE-OAA-SVM, the highest classification accuracy of 96.36% was obtained, which is an improvement of 13.33% over the model using only MRI data, as shown in Table 7. Overall, the introduction of image data significantly improved the classification performance, and the optimization effect of the differential evolution and improved differential evolution algorithms further enhanced the performance of the data fusion model. These results show that combining low-field NMR data with image data and optimizing the model parameters using a differential evolution algorithm can significantly improve classification accuracy, demonstrating the importance and potential of data fusion and optimization algorithms in this task.

**Figure 9:**
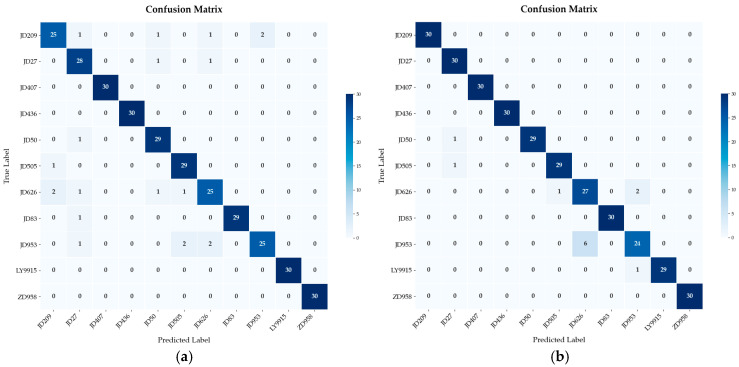
Model confusion matrix visualization. (**a**) DE-OAA-SVM model confusion matrix visualization. (**b**) HDE-OAA-SVM model confusion matrix visualization.

The HDE-OAA-SVM model performs well in classifying most of the varieties, with 100% classification accuracy for JD209, JD27, JD407, JD436, JD83, and ZD958, which indicates that the model is extremely robust in distinguishing these categories. However, there is still some classification confusion between some of the categories. For example, one sample each of JD50 and JD505 was misclassified as each other, probably due to their high similarity in crude protein (9.51% vs. 9.59%), crude fat (4.31% vs. 4.70%), and crude starch (72.6% vs. 73.27%), which made it difficult for the model to distinguish between them completely. Two out of three samples of JD626 were misclassified as JD953, one sample as JD953, one sample as JD953, and one as JD505, while six samples from JD953 were misclassified as JD626. This bi-directional confounding may be due to the proximity of the two samples in terms of crude protein (8.66% vs. 8.81%), crude fat (3.99% vs. 3.67%), and lysine (0.27% vs. 0.25%) contents, although there was some difference in crude starch (75.62% vs. 0.25%) and crude starch content. There were some differences in crude starch content (75.62% vs. 77.33%). In addition, one sample from LY9915 was misclassified as JD953, but despite these confounds, the model overall performed robustly, with high classification accuracy. Figure 9 illustrates a comparison of the confusion matrices of the model before and after the differential evolution improvement.

**Figure 10:**
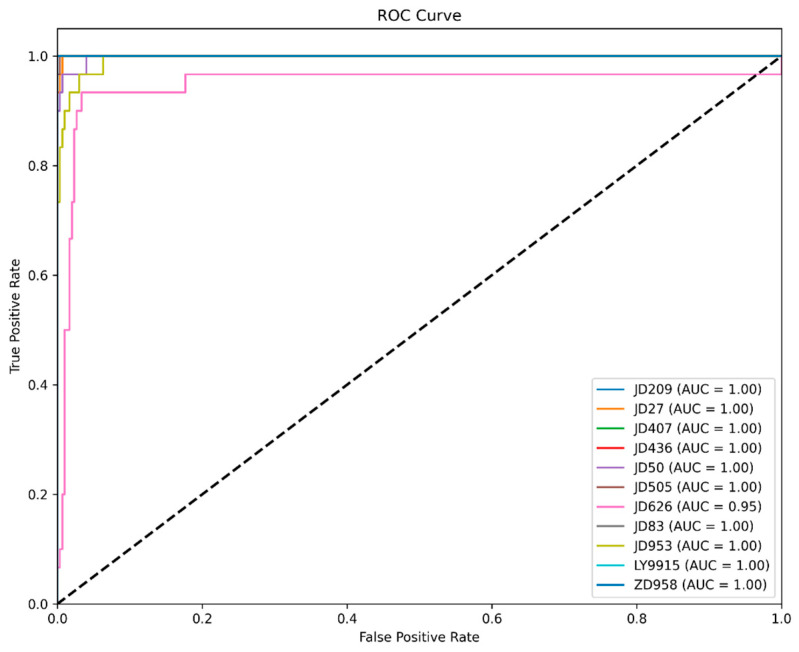
ROC curve visualization.

The AUC (area under the curve) value under the ROC curve is an important indicator for assessing the overall classification effect of the model, and the closer the AUC value is to 1.0, the better the classification performance is. The AUC values of most varieties (e.g., JD209, JD27, JD407, JD436, JD50, JD505, JD83, LY9915, ZD958) reach 1.00, indicating that the model’s classification performance on these varieties is extremely good, and accurate differentiation can be achieved. However, the AUC value of JD626 is 0.95, which is slightly lower than the other varieties, showing that there is some confusion in the model in distinguishing JD626 from varieties with similar characteristics (e.g., JD953), which is consistent with the results of the confusion matrix analysis, as shown in Figure 10. Overall, the ROC curve is close to the upper left corner, indicating that the model has a high true-positive rate and a low false positive rate, and the classification performance is robust.

**Figure 11:**
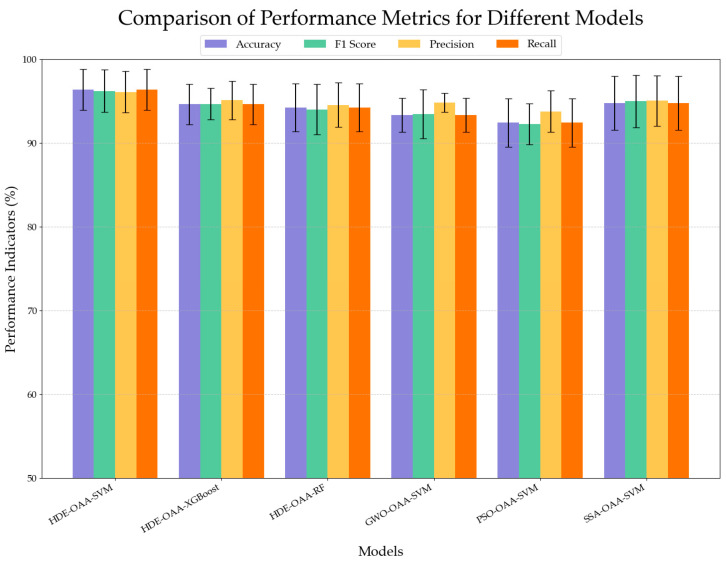
Performance comparison of different models.

In addition, this study applies the HDE method to the integrated learning models XGBoost and RF, respectively, with the aim of exploring the optimization effect of the HDE method in the integrated models. The advantages of HDE in terms of optimization performance are further evaluated by comparing with gray wolf optimization (GWO) [45], particle swarm optimization (PSO) [46], and a sparrow search algorithm (SSA) [47]. As shown in Figure 11, before and after the application of the HDE method, the OAA-SVM model consistently demonstrates superior performance compared to the XGBoost and RF models. Moreover, the HDE-OAA-SVM significantly outperforms other optimization algorithms across all evaluation metrics, with notably smaller errors. This indicates that the HDE-OAA-SVM model exhibits enhanced stability and consistency, underscoring its robustness in optimization.

### 3.4. Model Interpretation

**Figure 12:**
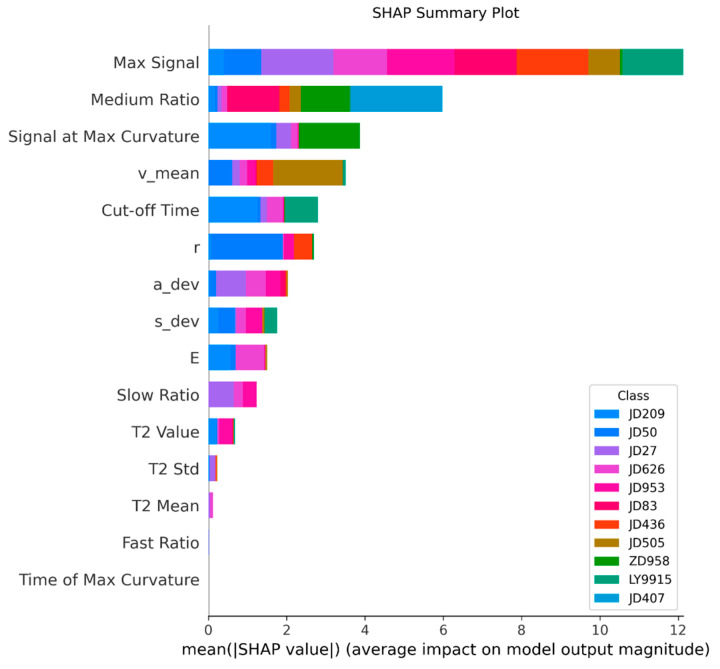
SHAP summary plot.

SHAP (Shapley additive explanations) is a game-theory-based explanation method for interpreting the prediction results of machine learning models. The SHAP summary plot reveals the importance of different features in the prediction of the HDE-OAA-SVM model and clarifies the average contribution of each feature to the classification of different corn varieties. As shown in Figure 12, Max Signal is the most important feature, with a significant impact on the classification of all varieties; the JD83 had the highest Max Signal value (79,038.033 ± 78.160 a.u.), which corresponded to its high crude protein content (10.92%), while the ZD958 had the lowest Max Signal (63,720.533 ± 61.114 a.u.), which is consistent with its lower crude protein content (8.47%). In addition, Medium Ratio and Signal at Max Curvature follow closely behind, showing high importance in the classification of varieties such as JD27, JD50, JD505, LY9915, etc. This correlates with their chemical fraction characteristics: JD50 and JD505 had similar crude protein contents (9.51% and 9.59%, respectively), but differed in crude fat content (4.31% and 4.70%, respectively). Although the overall contribution of secondary features such as Cut-off Time and ‘v_mean’ is low, they play a unique role in the classification of specific varieties such as JD209 and JD505. This multidimensional analysis not only revealed the decision-making mechanism of the model but also provided new perspectives for understanding the differences in seed chemical fractions.

SHAP analysis of JD27 confirmed the hierarchical importance of features in classification. Maximum Signal demonstrated the highest predictive power, while ‘a_dev’ and Slow Ratio served as significant complementary indicators. Notable contributions were also observed from Signal at Max Curvature, particularly at higher values. Although Cut-off Time and Medium Ratio exhibited relatively minor effects, they provided auxiliary discriminative information for specific samples. These findings suggest that the model’s effectiveness stems from its integration of both dominant NMR signatures and subtle morphological variations, enabling robust varietal discrimination.

**Figure 13:**
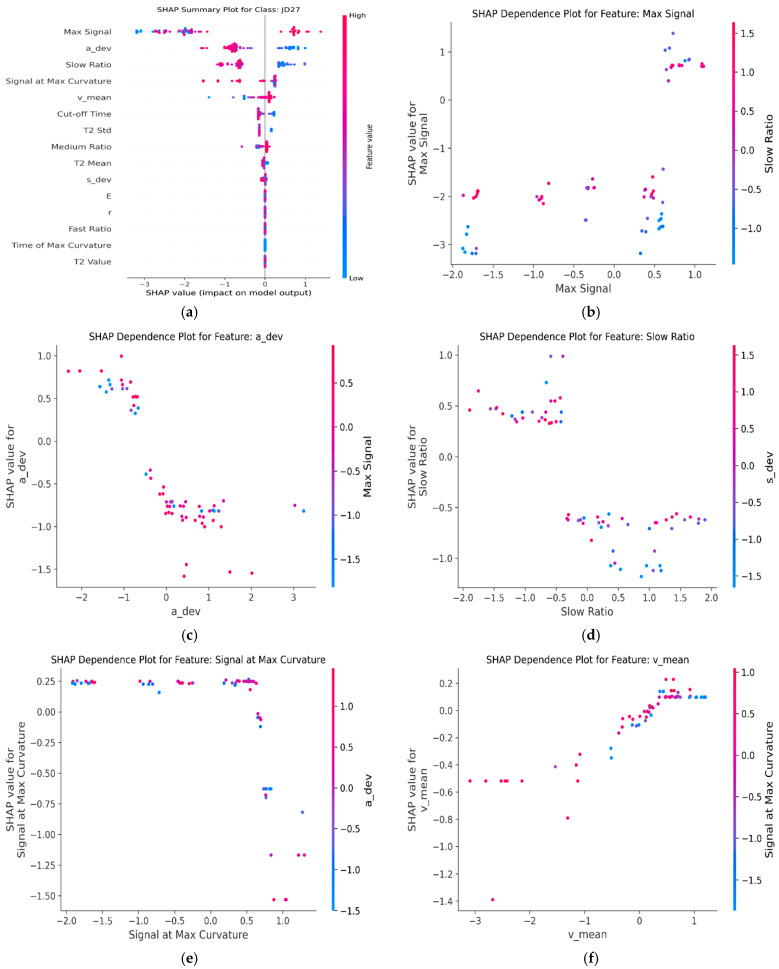
Shapley explanation for JD27: (**a**) summary plot of JD27; (**b**–**f**) SHAP dependence plots for Max Signal, a_dev, Slow Ratio, Signal at Max Curvature, and v_mean.

In the SHAP dependence analysis of JD27, the contribution of multiple features to the classification results of the model shows a clear nonlinear pattern and interaction, as shown in Figure 13. The contribution of Max Signal to classification shows a clear nonlinear pattern, with a significant increase in the positive contribution to classification in the high value region (>0.5). At the same time, a high value of Slow Ratio (>1.0) further amplifies the positive contribution, indicating that the synergistic effect of these two features plays a key role in model classification. This is consistent with the relatively low crude protein content (8.46%) and moderate crude fat content (4.06%) characterizing JD27. The contribution of ‘a_dev’ to classification decreases as its value increases. A low ‘a_dev’ value under high Max Signal conditions is more beneficial for classification, indicating a significant interaction effect of this feature in regulating classification. The segmented pattern of Slow Ratio reveals its negative contribution to classification in the low value region, while the positive contribution of the high value region (>1.0) is significantly enhanced, and it shows stronger classification ability under high ‘s_dev’ conditions. Signal at Max Curvature has a more significant positive contribution in the low value region (<0.5), and’ a_dev’ has a moderating effect on its contribution, in which the low ‘a_dev’ value (blue) has a more prominent positive contribution. In addition, the positive contribution of ‘v_mean’ gradually increases with the increase in the value, and it plays a more significant role under the condition of high Signal at Max Curvature, which shows the synergistic relationship between the two. Overall, the discrimination of JD27 depends on the synergistic effect of multiple features. These features not only have independent main effects, but also jointly optimize the classification performance of the model through significant interactions. This result provides an in-depth scientific basis for understanding the decision-making mechanism of the model and also emphasizes the importance of complex feature interactions in model interpretability.

## 4. Discussion

The practical value of this study extends beyond theoretical research to real-world applications, particularly in germplasm resource management and breeding programs. In seed bank quality control, our method offers a rapid, non-destructive alternative to traditional approaches such as DNA markers or protein electrophoresis, which often require sample destruction. This method demonstrates strong discriminatory power, especially for varieties with similar chemical compositions. For instance, it accurately distinguishes between JD50 and JD505, despite their closely comparable levels of crude protein (9.51% vs. 9.59%), crude fat (4.31% vs. 4.70%), and crude starch (72.6% vs. 73.27%). The integration of LF-NMR features enables the detection of subtle differences in internal composition. This is exemplified by the identification of varieties with distinct crude protein levels, such as JD436 (10.65%) and JD27 (8.46%). Furthermore, the method provides valuable support for hybrid breeding programs by characterizing germplasm resources based on their chemical profiles. For example, varieties with complementary traits can be efficiently identified: JD436 and JD407 both exhibit high starch content (76.39% and 76.60%, respectively), while LY9915 shows elevated crude fat content (4.99%). This precise characterization facilitates the selection of parent lines with desirable complementary traits, aiding in the development of improved hybrid varieties.

LF-NMR technology has shown great potential in many fields due to its rapid, non-destructive, and highly sensitive nature [48]. This study shows that combining LF-NMR data with image data not only enables accurate identification but also provides a direct basis for chemical composition analysis. LF-NMR data can provide information on the chemical characteristics of maize seeds, including hydrogen nuclear relaxation times and moisture distribution, which are closely related to seed quality indicators such as protein, fat and starch content. The introduction of image data complements the morphological information of maize seeds, such as color, size, and texture features. At the same time, SHAP analysis reveals the contribution of multiple key features to the classification results and provides a basis for interpreting the chemical background of these features. The methods in this study can be extended to the quality assessment and classification of other grains, and can also be used for the detection of food contaminants such as mycotoxins or heavy metals [49], achieving comprehensive coverage from classification to safety assessment.

The introduction of an optimization algorithm further exploits the potential of LF-NMR data. Through the differential evolution algorithm and its improved strategy, the classification accuracy of the model is improved from 89.39% to 96.36%. This performance improvement not only reflects the important role of the optimization algorithm in feature fusion but also shows that the synergistic effect of chemical features and image features can significantly improve the classification effect. In addition, the research results reveal the potential of LF-NMR data for chemical characterization. In the future, the dimensionality of chemical information can be further enriched by introducing more chemical analysis indicators (such as T1 relaxation time or chemical shift data), thereby improving the adaptability of the model in diverse scenarios [50].

Although this study has made significant progress in classification performance, there are still some limitations. The sample size is relatively small, which may limit the generalization ability of the model. In the future, more samples need to be introduced to cover seeds from different sources in order to enhance the stability and applicability of the model. The current research mainly focuses on the identification task, and the exploration of quantitative prediction of composition or multi-objective tasks still needs to be further studied.

## 5. Conclusions

This study proposes a maize kernel variety identification method that integrates LF-NMR data with image data. The model is optimized by enhancing the differential evolution algorithm to improve identification efficiency. This approach is particularly suited to the practical needs of germplasm resource optimization and agricultural production. Through PCA, this study reveals the close relationship between LF-NMR characteristics and internal components (such as crude protein, crude fat, starch, and lysine content), further clarifying the characteristic distribution of different varieties and their clustering trends. The experimental results show that data fusion significantly improves the performance of the classification model. Compared with using only LF-NMR data, the classification accuracy is improved by 6.36 percentage points to 89.39%. By further optimizing the model parameters through the differential evolution algorithm, the accuracy is improved to 93.94%. The introduction of an improved differential evolution algorithm further improves the accuracy to 96.36%, which is 13.33 percentage points higher than the single data source and achieves the optimal performance. In addition, Shapley additive interpretation was used to explore the feature importance and dependence in depth, revealing the positive impact of key features such as Max Signal, Medium ratio, Signal at Max Curvature, ‘v_mean’, and Cut-off Time on the discrimination performance and their interactions, which strengthens the interpretability of the model.

In summary, this study highlights the potential of integrating multi-source data fusion and optimization algorithms to enhance the performance of maize variety identification. It also provides valuable insights and scientific support for the application of LF-NMR data in the field of agriculture. Future research should focus on further refining feature selection and optimization strategies for multimodal data, as well as conducting more in-depth analysis of internal components. These efforts will help improve the model’s generalization ability and practical applicability, thereby advancing agricultural management and crop breeding.

## Figures and Tables

**Table 1:** The results of different group intelligence algorithms optimizing machine learning hyperparameters.

Model	Swarm Intelligence	Without Optimization (%)	With Optimization (%)	Ref.
AdaBoost	DE	91.00	98.80	Gupta et al. [36]
ELM	SSA	84.70	96.70	Shao et al. [37]
SVM	PSO	90.83	94.32	Tian et al. [38]
XGBoost	GA	93.25	94.82	Li et al. [39]
MASK-RCNN	PSO + SSO	95.7	97.67	Sudha et al. [40]

**Table 2:** Internal chemical composition of 11 maize varieties.

Variety	Crude Protein Content (%)	Crude Fat Content (%)	Crude Starch (%)	Lysine Content (%)
JD436	10.65	3.57	76.39	0.26
JD50	9.51	4.31	72.6	0.32
JD505	9.59	4.70	73.27	0.30
JD83	10.92	3.66	73.62	0.30
JD209	10.02	4.55	68.50	-
JD407	10.03	3.23	76.6	0.26
JD27	8.46	4.06	75.23	0.27
JD626	8.66	3.99	75.62	0.27
JD953	8.81	3.67	77.33	0.25
ZD958	8.47	3.92	73.42	0.37
LY9915	10.58	4.99	73.30	0.29

**Table 3:** Pseudo-code for HDE.

**Input:** Population size NP, Number of generations G_max, Initial mutation factor F_base, Initial crossover rate CR_base, Diversity adjustment parameters ∆F, ∆CR, Maximum diversity D_max.
1: Initialize population P(t) with NP individuals 2: Initialize base mutation factor F_base and crossover rate CR_base 3: for t = 1 to G_max do 4: Calculate current diversity D_Current of population P(t) 5: 6: Adjust F and CR based on convergence indicator c_t: 7: F_(t+1) = dynamic adjustment based on c_t and D_Current 8: CR_(t+1) = dynamic adjustment based on c_t and D_Current 9: for each individual i in P(t) do 10: Select mutation strategy based on optimization stage: 11: v_i^(t+1) = mutation using selected strategy 12: 13: Perform crossover to generate trial vector U_i 14: Select the better between U_i and X_i to form new population 15: end for 16: 17: Update and monitor the best solution 18: end for 19: 20: Return the best solution found

**Table 4:** Ten features of different types of maize kernels extracted from decay curves.

Variety	Max Signal	T_2_ Value	Time of Max Curvature	Signal at Max Curvature	Cut-Off Time	Fast Ratio	Medium Ratio	Slow Ratio	T_2_ Mean	T_2_ Std
JD209	76,705.667 ± 32.226	102.949 ± 0.732	0.6 ± 0.0	54,524.433 ± 68.615	477.347 ± 2.673	0.35 6 ± 0.002	0.412 ± 0.000	0.232 ± 0.001	0.043 ± 0.001	3.388 ± 0.011
JD27	77,147.600 ± 34.574	102.685 ± 0.513	0.6 ± 0.0	54,069.833 ± 92.314	477.80 6 ± 2.431	0.357 ± 0.001	0.413 ± 0.002	0.230 ± 0.001	0.043 ± 0.001	3.371 ± 0.008
JD407	71,997.300 ± 52.692	106.168 ± 0.704	0.6 ± 0.0	49,657.800 ± 99.009	458.180 ± 2.513	0.345 ± 0.001	0.408 ± 0.001	0.247 ± 0.001	0.045 ± 0.001	3.507 ± 0.010
JD436	63,854.300 ± 75.879	116.304 ± 0.891	0.6 ± 0.0	42,004.433 ± 122.864	522.460 ± 3.735	0.332 ± 0.002	0.413 ± 0.003	0.255 ± 0.002	0.04 6 ± 0.001	3.560 ± 0.012
JD50	76,939.967 ± 56.369	106.620 ± 0.805	0.6 ± 0.0	54,828.867 ± 112.201	503.913 ± 3.355	0.349 ± 0.002	0.413 ± 0.002	0.238 ± 0.001	0.042 ± 0.002	3.435 ± 0.011
JD505	77,511.633 ± 52.642	111.208 ± 0.871	0.6 ± 0.0	54,910.600 ± 103.264	510.927 ± 3.518	0.340 ± 0.002	0.411 ± 0.003	0.249 ± 0.002	0.04 6 ± 0.002	3.517 ± 0.012
JD626	76,564.633 ± 680.551	106.677 ± 0.793	0.6 ± 0.0	53,943.533 ± 510.497	493.167 ± 3.251	0.350 ± 0.002	0.413 ± 0.001	0.238 ± 0.002	0.044 ± 0.002	3.435 ± 0.012
JD83	79,038.033 ± 78.160	110.852 ± 0.974	0.6 ± 0.0	57,131.967 ± 121.908	529.487 ± 4.144	0.343 ± 0.002	0.415 ± 0.001	0.242 ± 0.002	0.045 ± 0.002	3.461 ± 0.014
JD953	75,687.700 ± 52.365	112.292 ± 0.889	0.6 ± 0.0	53,230.867 ± 109.357	516.640 ± 3.589	0.339 ± 0.002	0.413 ± 0.002	0.248 ± 0.002	0.04 6 ± 0.002	3.512 ± 0.012
LY9915	68,638.200 ± 49.359	117.512 ± 0.826	0.6 ± 0.0	46,792.267 ± 85.680	561.973 ± 3.623	0.332 ± 0.001	0.414 ± 0.002	0.254 ± 0.001	0.04 6 ± 0.001	3.558 ± 0.011
ZD958	63,720.533 ± 61.114	104.125 ± 0.405	0.6 ± 0.0	41,668.567 ± 84.462	485.087 ± 2.035	0.35 6 ± 0.001	0.415 ± 0.002	0.229 ± 0.001	0.043 ± 0.001	3.362 ± 0.007

**Table 5:** Comparison of classification performance using full feature set and reduced feature set.

Model	Accuracy Mean (%)	Accuracy Std (%)	F1 Score Mean (%)	F1 Score Std (%)	Precision Mean (%)	Precision Std (%)	Recall Mean (%)	Recall Std (%)
3000-feature	87.58	3.76	87.63	3.54	90.24	2.78	87.58	3.76
10-feature	83.03	3.54	81.88	4.41	86.26	2.51	83.03	3.54

**Table 6:** Comparison of multiple classification models.

Model	Accuracy Mean (%)	Accuracy Std (%)	F1 Score Mean (%)	F1 Score Std (%)	Precision Mean (%)	Precision Std (%)	Recall Mean (%)	Recall Std (%)
OAA-SVM	89.39	3.95	89.53	4.18	92.44	2.47	89.39	3.95
Logistic Regression	87.88	4.18	88.26	4.27	91.54	3.21	87.88	4.18
Random Forest	86.67	3.37	86.83	3.66	89.63	3.94	86.67	3.37
K-Nearest Neighbors	82.42	3.26	82.44	3.13	85.64	2.65	82.42	3.26
MLP Classifier	88.48	3.4	88.81	3.17	91.03	2.47	88.48	3.4
XGBoost	88.79	1.82	89.11	1.45	91.39	1.01	88.79	1.82

**Table 7:** Performance comparison of OAA-SVM and improved model in maize kernel classification.

Set	JD209	JD27	JD407	JD436	JD50	JD505	JD626	JD83	JD953	LY9915	ZD958	Cross-Validation Accuracy (%)
Training sets (*n* = 264)	24	24	24	24	24	24	24	24	24	24	24	/
Verification sets (*n* = 66)	6	6	6	6	6	6	6	6	6	6	6	/
Only image	31	36	25	36	33	34	39	11	24	24	37	69.09 ± 3.66
Only LF-NMR	21	31	30	30	29	41	22	35	29	32	30	83.03 ± 3.54
Image + LF-NMR	29	33	30	30	37	37	23	31	20	30	30	89.39 ± 3.95
DE-OAA-SVM	28	33	30	30	32	32	29	29	27	30	30	93.94 ± 3.46
HDE-OAA-SVM	30	32	30	30	29	30	33	30	27	29	30	96.36 ± 2.45

Note: ‘*n*’ is the number of samples and ‘/’ denotes the null value.

## Data Availability

Data are available from the author upon reasonable request.
